# Points in Three London General Hospitals

**Published:** 1907-06-08

**Authors:** 


### POINTS IN THREE LONDON GENERAL HOSPITALS.

### The Metropolitan Hospital.

Situated in one of the poorest and most densely populated districts of London, *i.e.*, Kingsland Road, E., this hospital has never yet succeeded in attracting all the financial support which it needs. It is an institution which should have the special consideration and monetary aid of the Jewish community, to the poorer portion of which it ministers with prodigal generosity. The number of in-patients under treatment shows a steady development, having grown from 1,131 in 1901 to 1,548 in 1906. The average daily number of beds occupied has increased by 25 per cent. in the five years—namely, from 86 to 106. The average cost of each in-patient as stated by the hospital authorities has fallen from £1 18s. 3d. to £1 14s. 1d., an evidence of the successful efforts made by the Committee and officers to reduce expenditure. The out-patients continue to increase, to our regret, but the work of the almoner has been vigorously continued with a view to reduce the abuse of this department to a minimum. This hospital is worthy of the attention of the benevolent, from the circumstance that it has a provident department which affords the working classes in its neighbourhood an opportunity to secure adequate medical treatment to themselves and their families when they are ill by small periodical payments. Out of 41,642 new out-patient cases 3,469 were referred to the provident department or to provident dispensaries during 1906. We could have wished that the number so referred had been considerably larger. Lord Howard de Walden, as Chairman, takes an active interest in the work of the hospital, and we have little doubt that under his guidance increased financial support, additional economy, and further efficiency in the administration will be manifest each year.

### The Middlesex Hospital.

The Middlesex Hospital is entitled to receive the generous support not only of Londoners but of the country generally, from the circumstance that it has maintained an in-patient department for cancer cases since the date of its foundation in 1745. It can proudly claim to be the first hospital in the British Islands which made special provision for the treatment of cancer. The administration has always been conservative, but in recent years it has displayed a progressive spirit, and it can claim that its numerous departments are as efficient and ably administered as any in London. Situated as it is in the centre of probably the wealthiest class of traders in London, *i.e.*, the great shopkeepers of Regent Street, Oxford Street, and the neighbourhood, we have always felt that an active propaganda on behalf of the institution should speedily make it the best supported of voluntary hospitals. There is a growing feeling in the medical profession that it is wrong for a great commercial emporium to give a relatively small subscription to a voluntary hospital, and then to claim in-patient accommodation free of all cost for every one of its employees who may need such accommodation in the course of each year. Middlesex Hospital might well adopt the plan which has proved successful with the few hospitals which have tried it, of rendering an account each year to the employer setting forth the actual cost to the hospital of the treatment of their employees, the actual subscriptions received from such firms, and the amount due to the hospital from those firms, as represented by the difference between these two sums. We believe that if the Middlesex Hospital authorities were to appoint an Economy and Finance Committee to go fully into this matter, and to work such a scheme thoroughly, they would find that the huge difference of £10,000 a year, by which their total ordinary expenditure exceeded their total ordinary income in 1906, would speedily disappear. Meanwhile it is satisfactory to note that the Committee are able to claim from the work of the Economic Committee that extensive economies have recently been effected which must reduce the cost per bed very materially.

### St. Mary's Hospital.

St. Mary's Hospital is the great general hospital of West London, and for this reason it should command a large income from the wealthier residents of the metropolis. Annual subscriptions are the backbone of voluntary hospital finance. One test of the efficiency of the management in the case of St. Mary's Hospital in the circumstances named must be the actual sum received each year from subscriptions. The average number of beds daily occupied in 1906 at the Middlesex Hospital was 266.5, and at St. Mary's Hospital 253.2. The amount received in annual subscriptions by the Middlesex Hospital was £2,871, and by St. Mary's Hospital £5,018. These figures show that the business management of St. Mary's Hospital in this respect is good. Yet the support received from West London by St. Mary's Hospital is at present so inadequate that the Clarence Wing, recently erected with accommodation for 60 beds, remains unfurnished and unoccupied, although we can find no allusion to this fact in the annual report just issued. But the hospital authorities state that they are compelled to deny admission daily to poor sick people living in the district, owing to an insufficiency of beds. We feel this state of things to be one of the most serious facts in the hospital situation this year, and we venture to hope that the managers will actively exert themselves, for then we have little doubt that all the money needed will be forthcoming from the great wealthy population to be found within a three miles radius of St. Mary's Hospital. At the present moment economy is the one feature which hospital supporters regard with the closest attention, and St. Mary's Hospital claims that the average cost per occupied bed—under £84—is almost the lowest among the general hospitals of London. This one fact, if driven home with sufficient reiteration and energy, should produce all the money needed to bring the sixty unoccupied beds into use, and to place St. Mary's Hospital once and for all in a strong financial position.

